# Experimental Research on Fluid Coupling Flexible Actuator

**DOI:** 10.3390/mi9030103

**Published:** 2018-02-28

**Authors:** Xiangli Zeng, Yue Wu, Qianjin Tu, Jingshi Dong, Zhigang Yang, Xinbo Li

**Affiliations:** 1 College of Mechanical Science and Engineering, Jilin University, Changchun 130025, China; xlzeng17@mails.jlu.edu.cn (X.Z.); tuqj14@mails.jlu.edu.cn (Q.T.); dongjs@jlu.edu.cn (J.D.); yzg@jlu.edu.cn (Z.Y); 2 College of Communication Engineering, Jilin University, Changchun 130025, China; xb_li@jlu.edu.cn

**Keywords:** piezoelectric, flexible actuator, fluid-solid coupling, displacement amplification, flexible diaphragm

## Abstract

In the field of micromechanics, piezoelectric actuator has attracted great attention for its high-frequency response, high displacement resolution, and high output force. However, its prospect of practical application has been largely limited by the displacement of micrometer. A fluid coupling flexible actuator was proposed, which utilizes resonance to enlarge the output displacement. The actuator uses a piezoelectric oscillator as an excitation source, fluid as the transmission medium and a flexible diaphragm for the displacement output. On the condition that the fluid is inviscid and incompressible, mathematical formulation of the membrane vibration theory has been analyzed. Then, the prototype is made. The displacement is amplified 21 times to 1.106 mm when driving frequency is 127 Hz. The flexible diaphragm appears the largest displacement output when driving frequency is close to one of the system’s natural frequency. Then, the points with zero amplitude form a circle on the surface of flexible diaphragm and the movement direction of the flexible diaphragm is opposite on different sides of the circle. In fact, rather than vibrates at the first resonance frequency, the membrane in the essay is vibrating at a certain higher-order resonance frequency. The experimental results are mainly consistent with the theoretical analysis.

## 1. Introduction

A variety of smart driving materials applied in the field of micro-electromechanical systems (MEMS) have been developed. Comparing with shape memory alloys (SMA) and electroactive polymers (EAP) [1], piezoelectric materials have the superiorities like faster response, larger output force, higher energy density, higher bandwidth, more simple structure, and lower power consumption [2,3]. However, the micron level displacement output limits its scope of application, where the displacement amplification mechanism is a major research orientation. Hitherto there are two common categories of displacement amplifiers: One is stack or leverage mechanical amplification mechanism [4]. Although these agencies can achieve displacement amplification, their structure is complex and occupies a large space. The other is hydraulic amplification mechanism that produces displacement amplification through an incompressible fluid. The fluid is sealed between two pistons with different areas [5]. These structures produce bigger displacement output with less space comparing to the mechanical amplifier.

Numerous fluidic mechanisms that use hydraulic amplification units have been developed to amplify the displacement of piezoelectric actuators. Yoon et al. studied the displacement characteristics of piezoelectric micro actuator based on hydraulic amplification system [6,7]. Roberts et al. applied the hydraulic amplification structure to the micro valve field, and studied its output performance [8]; Many scholars also studied the tactile feedback system that is based on the hydrostatic coupling flexible structure [9,10]; Ninomiya et al. used hydraulic amplification mechanism to amplify the piezoelectric micro displacement and the output by a flexible diaphragm for Braille display [11], the results show that the static and dynamic characteristics of hydraulic amplification are different, and the amplification is more obvious at the resonance frequency point [12]. Forming displacement output capability by combining the flexible diaphragm and liquid shows a good application prospect in human-computer interaction system. But, as a complex system, including fluid, solid, and elastomer, it applies only on the premise that research characteristics of the system more further. Therefore, this paper proposes a fluid coupling flexible actuator based on hydraulic amplification mechanism and membrane vibration theory, which uses a piezoelectric oscillator to excite the system resonance and cause the flexible diaphragm deformation. The bigger displacement output appears at the center of the flexible diaphragm and it has the ability to amplify the output displacement of the piezoelectric oscillator. The mathematical formulations of the free vibration of a circular membrane were analyzed. Then, the experimental device was developed and the output performance was tested. When comparing with the traditional stack or leverage mechanical amplification mechanism, a new method to achieve amplification is proposed and tested by experiment. For providing a bigger amplification ratio, the system proposed could combine with the conventional hydraulic amplifier by changing the areas of membrane and oscillator.

## 2. Actuator and Its Working Principles

### 2.1. The Structure of the Actuator

**Figure 1:**
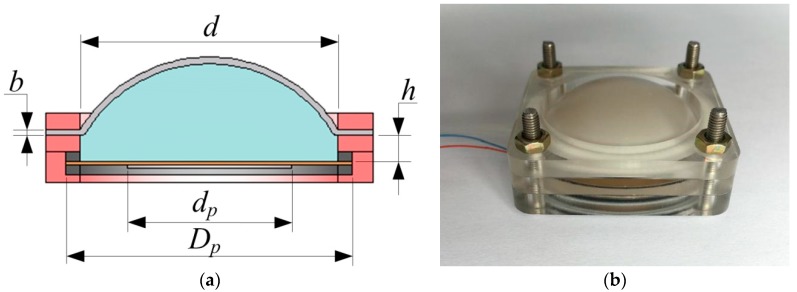
The designed actuator. (**a**) Structure diagram of the actuator; (**b**) The actuator prototype.

This paper proposes a novel fluid coupling piezoelectric actuator. Actuator, which is shown in Figure 1a, includes a cavity shell, a piezoelectric oscillator as an excitation source, incompressible fluid as power transmission medium and a flexible diaphragm as the displacement output. There is a cylindrical chamber inside of the shell, whose diameter is represented by *d*. The piezoelectric oscillator consists of a circular copper substrate and a piezoelectric wafer and the diameter of the copper substrate (*D_p_*) is 35 mm, the diameter of the piezoelectric wafer (*d_p_*) is 25 mm. For this actuator structure, the upper and lower surfaces of the chamber are respectively used a flexible diaphragm and a piezoelectric oscillator. Water was chosen as the transmission medium. Silicone diaphragm was selected as the flexible diaphragm, which is cut from a large piece of silicone membrane for the reason that such silicone diaphragm has high elasticity and flexibility. A new silicone diaphragm was used in each experiment to avoid the change of the performance parameters that is caused by excessive stretching. Here, the effective diameter of the piezoelectric oscillator is the same as the silicone diaphragm. The thickness of the silicone diaphragm is represented by *b*. The chamber height (*h*) refers to the distance from the top surface of the piezoelectric vibrator to the lower surface of the clamping portion of the silicone diaphragm. Meanwhile, there is a liquid injection channel in the side of chamber. Excitation produced by the piezoelectric oscillator through the incompressible fluid acts on the silicone diaphragm forming the ability of displacement output. The proposed actuator prototype is shown in the following Figure 1b.

Except for special statement, this paper adopts the parameters are listed in Table 1:

### 2.2. Working Principle

**Figure 2:**
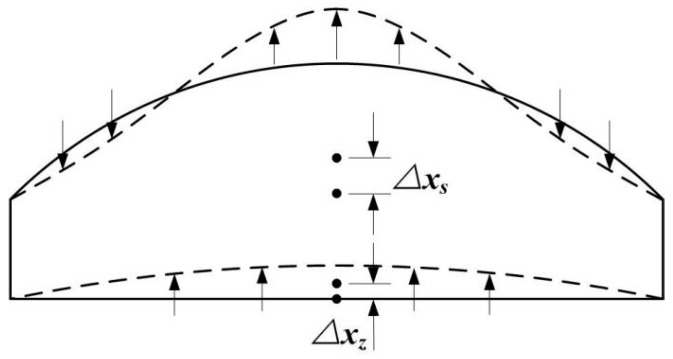
The deformation diagram of the silicone diaphragm.

The working process of the prototype is as follows. The piezoelectric oscillator driven by alternating voltage provides reciprocating bending deformation. The energy through the incompressible fluid in the chamber drives silicone diaphragm moving up and so that forms the ability of displacement output. In the situation that the silicone diaphragm is working at the resonant frequency, a circle whose points with zero amplitude appears on the surface of the silicone diaphragm. The motion of the fluid on different sides of the circle is opposite. Because the fluid cannot be compressed, it will enlarge the output displacement on the center point of silicone diaphragm, as shown in Figure 2, and more proofs of this conclusion will be discussed in detail in Section 4.3. Two indicators, which indicate the actuator’s amplification ability, have been discussed. One indicator that evaluates actuator displacement amplification discussed in this article is the output displacement of the center of silicone diaphragm. It can directly reflect the actuator’s output capacity. The other indicator is amplification ratio, which is defined as the ratio between the midpoint output amplitude of the silicone diaphragm and the midpoint input amplitude of the piezoelectric oscillator. Both of them can easily display driver’s coupling characteristics; we can use them to identify the best working conditions as well as the amplification ability of the actuator.

## 3. Mathematical Formulation

Under the assumptions that the amplitudes of the piezoelectric oscillator and the fluid surface are both small amplitude and the fluid is considered to be inviscid and incompressible, the motion of the fluid is irrotational, below we can deduce mathematical formulations [13,14].

According to Hamilton’s Principle, we can reach the formulation of the vibration of the membrane by separating the variable *ω*(*r*, *θ*, *t*) = *ϕ*(*r*, *θ*)*q*(*t*).

(1) $$\frac{\partial^{2}w}{\partial t^{2}}=a^{2}\left|\frac{\partial^{2}\phi }{\partial r^{2}}+\frac{1}{r}\frac{\partial \phi }{\partial r}+\frac{1}{r^{2}}\frac{\partial^{2}\phi }{\partial \theta^{2}}\right|$$

In the formulation, *a*^2^ = *T*/*ρ* where the tension of membrane is shown by *T* and the mass per unit area of membrane is presented by *ρ*. The parameter *T* is related to the initial state and the thickness of the membrane. The radius of the membrane is presented by *l* and there are boundary conditions as follows:

*ω*(*l*, *θ*, *t*) = 0 (2)

*ω*(*r*, *θ*, *t*) = *ω*(*r*, 2π, *t*) (3)

It is easy for us to deduce differential Equations (4) and (5) from Formulation (1).

(4) $$\ddot{q}(t)+\omega^{2}q(t)=0$$

(5) $$\frac{\partial^{2}\phi }{\partial r^{2}}+\frac{1}{r}\frac{\partial \phi }{\partial r}+\frac{1}{r^{2}}\frac{\partial^{2}\phi }{\partial \theta^{2}}+\frac{w^{2}}{a^{2}}\phi =0$$

The solution of Equation (4) is shown, as follows:

*q*(*t*) = *C*_1_sin(*ωt* + *φ*) (6)

Then, by separating the variable *ϕ*(*r*, *θ*) = *R*(*r*)Ф(*θ*) the Equation (5) would be converted to be Equations (7) and (8).

(7) $$\frac{d^{2}\Phi }{d\theta^{2}}+n^{2}\theta =0$$

(8) $$\frac{r^{2}}{R}\frac{d^{2}R}{dr^{2}}+\frac{r}{R}\frac{dR}{dr}+r^{2}\lambda^{2}-n^{2}=0$$

in which *λ*^2^ = *ω*^2^/*a*^2^. Solution of Equation (7) is shown, as follows:

Ф(*θ*) = *C*_2_sin(*nθ* + *ψ*) (9)

In the Equation (9), *n* is supposed to be an integer because of the boundary condition Equation (3). After simplification, the Equation (8) could be changed into a No. *n* order Bessel function like Equation (10).

(10) $$r^{2}\frac{d^{2}R}{dr^{2}}+r\frac{dR}{dr}+(r^{2}\lambda^{2}-n^{2})R=0$$

Solution of Equation (10) is showed as follow:

*R*(*r*) = *C*_3_*J_n_*(*λr*) + *C*_4_*Y_n_*(*λr*) in which *C*_4_ = 0 for the reason that *Y_n_*(0) = ∞.

Finally, the solution of Equation (10) is as follows:

*R*(*r*) = *C*_3_*J_n_*(*λr*) (11)

*J_n_*(*l*) = 0 as a result of *R*(*l*) = 0 due to the boundary condition (2).

$\beta_{m}^{\left(n\right)}$ is signed to be the No. *m* null point of the No. *n* order Bessel function. Further, we can deduce the resonance frequency *ω_mn_*.

(12) $$\lambda_{m}^{\left(n\right)}l=\beta_{m}^{\left(n\right)},\omega_{mn}=\beta_{m}^{\left(n\right)}\frac{a}{l}$$

In conclusion, the solution of the free vibration of a circular membrane is given as follow:

(13) $$\omega (r,\;\theta ,\;t)=\sum_{m=1}\sum_{n=0}A_{mn}J_{n}(\beta_{m}^{\left(n\right)}\frac{r}{l})\sin(n\theta +\psi )\sin(\omega_{mn}t+\varphi )$$

**Figure 3:**
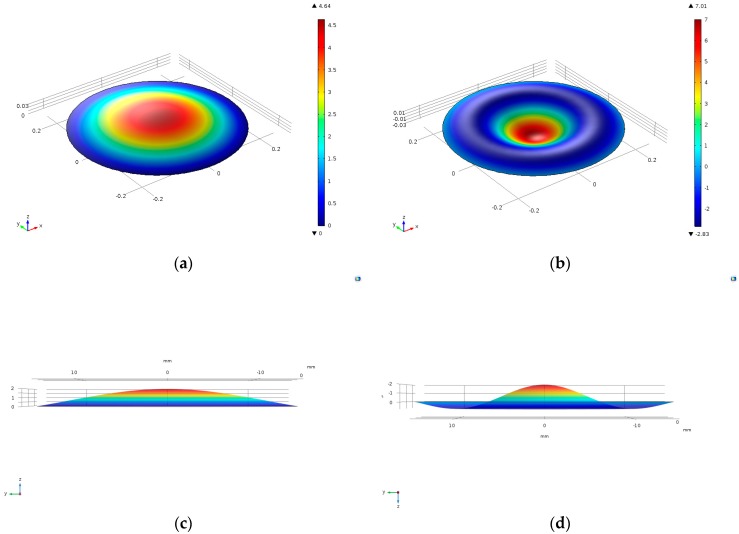
(**a**) Film vibration image with *m* = 1, *n* = 0; (**b**) Film vibration image with *m* = 2, *n* = 0. (**c**) Sectional view along diameter with *m* = 1, *n* = 0; (**d**) Sectional view along diameter with *m* = 2, *n* = 0.

In order to show the vibration of the membrane more visual, we will give a breif graphic presentation with specific parameters that *l* = 250 mm, *T* = 20,000 N/m, *ρ* = 3.925 × 10^7^ kg/m^2^ in the case of *m* = 1, *n* = 0 and *m* = 2, *n* = 0, respectively, in Figure 3.

On the condition that *m* = 1, *n* = 0, the maximum of absolute value of the displacement is 4.64 m and is 7.01 m when *m* = 2, *n* = 0. By analyzing Figure 3a,b, we could conclude that membrane would appear bigger displacement if it vibrates at a certain higher order resonance frequency. Especially, in Figure 3b, there is a pitch circle in which the membrane has positive displacement and out which the membrane has negative displacement. Figure 3c,d are sectional views of vibrating membrane along diameter for showing the shape of membrane more clearly.

## 4. The Experimental Test and Discussion

### 4.1. Experimental Apparatus and Test Methods

**Figure 4:**
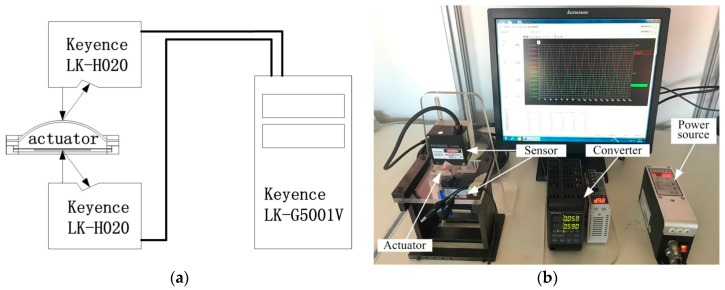
Experimental system of the designed actuator. (**a**) The layout of the sensor and the detection points; (**b**) Experimental setup for measuring the amplitude of the piezoelectric actuator.

In order to study the effect of various parameters on displacement amplification of actuator and to explore the frequency characteristics of the structure, the experimental prototype was developed. An actuator base was fixed on the support frame. Two laser displacement sensors (Keyence LK-H020, Keyence Corporation, Osaka, Japan) were arranged on different sides of the actuator to measure the amplitude of the center of silicone diaphragm and piezoelectric oscillator. The analog signal was converted to digital signal and stored through a controller (Keyence LK-G5001V, Keyence Corporation, Osaka, Japan), as shown in Figure 4a. Figure 4b shows a schematic view of the experiment. Sinusoidal AC power (SDVC40, Nanjing CUH Science & Technology Co., Ltd., Nanjing, China) was used to actuate piezoelectric oscillator. The non-contact measurement has the advantages of high accuracy, non-interference, and so on.

### 4.2. The Fluid Mass

**Figure 5:**
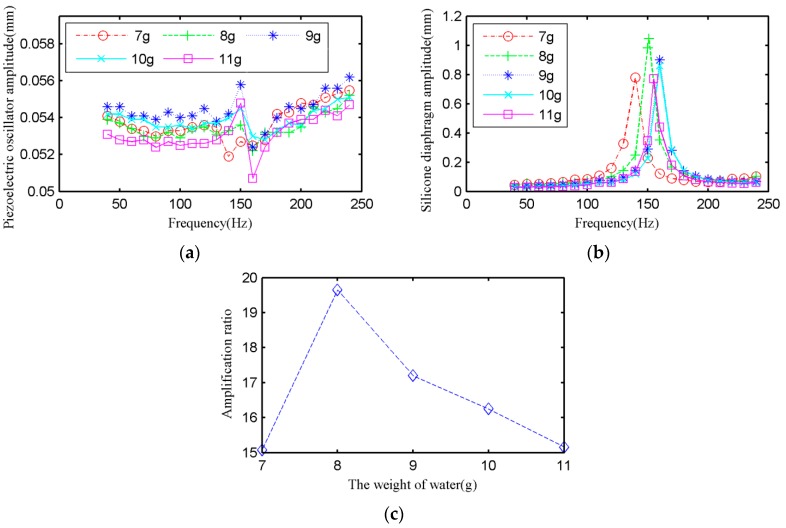
Experimental results: (**a**) The amplitude of piezoelectric oscillator under different water filling mass. (**b**) The amplitude of silicone diaphragm under different water filling mass. (**c**) The amplification ratio when the resonance under different water filling mass.

Fluid mass is one of the main parameters. In order to work out the most appropriate quantity of liquid, which will be used to conduct experiment further, water is selected as the fluid and the quantity of water are 7 g, 8 g, 9 g, 10 g, and 11 g. It should be noted that when the water is 7 grams, the silicone diaphragm has been expanded. The silicone diaphragm thickness is 0.5 mm. The results are shown in Figure 5.

Figure 5a shows the frequency characteristics of the midpoint displacement of the piezoelectric oscillator with different quantity of water. It can be seen that the maximum amplitude fluctuation of piezoelectric oscillator is less than 10%. The effect of fluid mass on the amplitude of the piezoelectric oscillator is slight, which is consistent with the law that the amplitude of the piezoelectric oscillator is basically unchanged when the quantity of water was increased in the dynamic model.

Figure 5b shows the frequency characteristics of the midpoint of the silicone diaphragm with a different quantity of water. We can see that with the increase of the water quantity, the resonance frequency increases first and then decreases. When the water quantity is 9 g or 10 g, the resonance frequency reaches 160 Hz. With the increase of the water quantity, the maximum amplitude of the center of the silicone diaphragm increases at first and then decreases at resonance. When the water quantity is 8 grams and the driving frequency is 151 Hz, the maximum amplitude of the silicone diaphragm reaches 1.047 mm.

Figure 5c shows the amplification ratio under the conditions of different water quantity. According to the experimental results, the amplification ratio of the system resonance increases at first and then decreases with the increase of the water quantity. The displacement amplification rises up to 19.65 times when the driving frequency is 151 Hz and the filling water quantity is 8 g.

### 4.3. The Thickness of the Silicone Diaphragm

To study how the silicone diaphragm thickness effects on the displacement amplification (silicone diaphragm displacement output and magnification ratio), four kinds of thickness of silicone diaphragm was contrasted: 0.3 mm, 0.5 mm, 1 mm, and 2 mm. 0.3 mm is the minimum value that the strength requirement is satisfied. The quantity of water is 8g as the result of research in 4.2. The silicone diaphragm is in a curved state.

**Figure 6:**
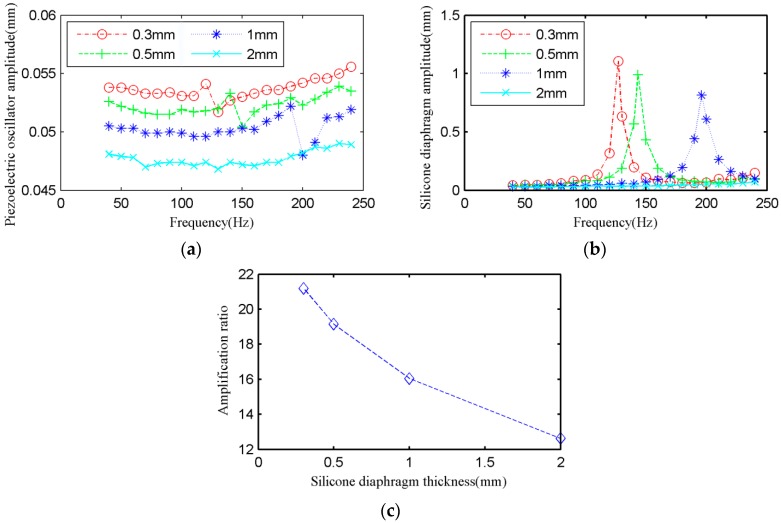
Experimental results: (**a**) The amplitude of piezoelectric oscillator under different silicone diaphragm thickness. (**b**) The amplitude of silicone diaphragm under different silicone diaphragm thickness. (**c**) The amplification ratio when the resonance under different silicone diaphragm thickness.

Figure 6a shows the frequency characteristics of the center displacement of piezoelectric oscillator. It can be seen that the amplitude of piezoelectric oscillator fluctuates in a range of 8%. The amplitude of piezoelectric oscillator decreases slightly with the increase of silicone diaphragm thickness (*b*).

Figure 6b shows the frequency characteristics of the midpoint displacement of silicone diaphragm. On the one hand, the frequency corresponding to the maximum amplitude increases with the increase of silicone diaphragm thickness (*b*). When the silicone diaphragm thickness is 2 mm, the system failed to resonate in the driving frequency range of 40–240 Hz. On the other hand, with the increase of silicone diaphragm thickness (*b*), the maximum amplitude of displacement output reduced. When the silicone diaphragm thickness is 0.3 mm and the driving frequency is 127 Hz, then the amplitude of the silicone diaphragm reached maximum 1.106 mm.

By combining Figure 6a,b, it can be seen that there is an inflection point that indicates the system resonates at this frequency on the cave of the displacement of piezoelectric oscillator, at the same time the silicone diaphragm has a maximum displacement.

Figure 6c shows that the maximum amplification ratio decreases with the increase of silicone diaphragm thickness (*b*). Therefore, it is concluded that the thinner the silicone diaphragm, the better the capacity of amplification during resonance. When the driving frequency is 127 Hz and the silicone diaphragm thickness is 0.3 mm, then the amplification ratio can reach 21.19 times, which is bigger than the traditional static hydraulic amplifier.

Numerical calculation and the experimental results are compared in the following. Theoretically, the parameter *ω_mn_* increases when the parameter *T* increases with the increase of the thickness of the Silicone diaphragm (*b*). The experimental results and theoretical analysis have the same trend.

Amplitude characteristics of the silicone diaphragm depend on its surface deformation, which will be discussed later in Section 4.4.

### 4.4. Experimental Verification

According to the above experimental results and analysis, the displacement output is remarkably enlarged. However, in theory, the vibration of silicone diaphragm is analyzed only. It would be much different if we take liquid into consideration on account that fluid-solid coupling may change the characteristics of both liquid and silicone diaphragm. In order to figure weather the experiment could meet theory completely, we test the surface deformation of the silicone diaphragm and conduct the simulation with a cambered membrane according to mathematic formulations in Section 3.

**Figure 7:**
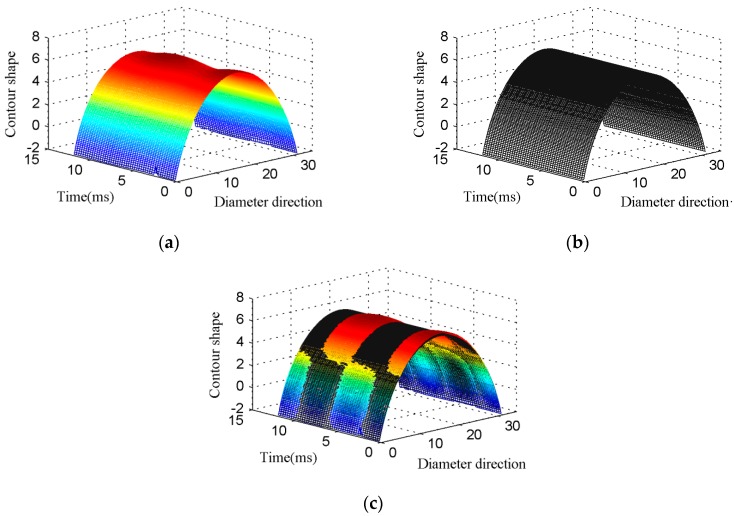
Experimental results: (**a**) The change of the cross-section profile of the silicone diaphragm during the dynamic work. (**b**) The cross-section profile of the silicone diaphragm at static. (**c**) Comparison of dynamic and static.

In order to measure the deformation, laser displacement sensor is used (Keyence LJ-V7080, Keyence Corporation, Osaka, Japan) to measure the vibration of each point along a diameter of the silicone diaphragm. The experimental parameters are listed, as follows: the thickness of silicone diaphragm (***b***) is 0.5 mm, the quantity of water (*m_s_*) is 8 g, and the drive frequency is 128 Hz. The test results are shown in Figure 7, the X-axis (Diameter direction) indicates the position of different points along the diameter of the silicone diaphragm, and the Z-axis (Contour shape) indicates the height of the different points on the silicone diaphragm, namely when the Y-axis is fixed, the curve in the XZ plane indicates the outer surface profile of the silicone diaphragm. The Y-axis (Time) coordinates represent the time of vibration, so the profile curve is transformed into a time-varying surface. Figure 7 contains a change process of 1.5 cycles of the section shape.

The change process of the outer surface profile of the silicone diaphragm during vibration is shown in Figure 7a (The colored surface), its stationary profile is shown in the Figure 7b (The black surface). In order to identify the dynamic process of the silicone diaphragm, the two surfaces are integrated, which is shown in Figure 7c. As illustrated in the figure, the colored surface in the center area is above the black surface, when the colored surface in the surrounding area is under the black surface, which is the central area of silicone diaphragm goes up when the peripheral area experiences go down. There is a special circle on the surface of silicone diaphragm during the vibration, the amplitude of each point on the circle is about zero, and the moving direction is opposite on different sides of this circle. Then, the centroid displacement of fluid is greater than the piezoelectric oscillator. The position of fluid centroid is directly affected by the shape of the silicone diaphragm, and this special deformation led to the fact that the midpoint displacement of the silicone diaphragm is bigger than the centroid displacement of the fluid, as shown in Figure 2. Generally speaking, the displacement amplification mechanism of the actuator is caused by the special deformation of the flexible diaphragm.

**Figure 8:**
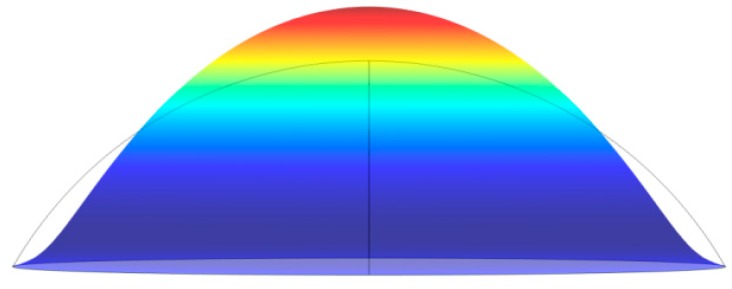
Image of simulation with a cambered membrane.

The simulation results are shown in Figure 8. The membrane in this simulation is cut from a circle whose radius is 15.75 mm. The grey line in Figure 8 represents the initial outline of membrane’s sectional view along the diameter and colored image shows the shape when the center point of the membrane reaches the biggest displacement.

Just as the experiment result concluded, there is a pitch circle with no displacement, which is shown by two points where the grey line and outline of colorful image overlaps in Figure 8. The part higher than grey line expresses the part with positive displacement in the experiment, in contrast, lower expresses the part with negative displacement. The outcome of simulation just meets the result of the experiment.

## 5. Conclusions

A fluid coupling flexible actuator was proposed to amplify the displacement of piezoelectric oscillator. A piezoelectric oscillator was used as an excitation source, fluid as the transmission medium, and a flexible diaphragm for the displacement output. The mathematical formulations of the free vibration of a circular membrane were analyzed, and then the frequency characteristics of the actuator can be predicted. It is generally acknowledged that the displacement would reach a bigger value when the system vibrates at the first resonance frequency, yet the membrane in the essay is vibrating at a certain higher-order resonance frequency. The displacement amplification ratio of the actuator is 21.19 times and the displacement output reaches 1.106 mm when the driving frequency is 127 Hz, the thickness of the silicone diaphragm is 0.3 mm, and the water quantity is 8 g. The points with zero amplitude form a circle on the surface of silicone diaphragm, and the directions of motion on the circle’s different sides are opposite. This phenomenon gives rise to an enhanced amplification of output displacement.

## Figures and Tables

**Table 1:** Parameters of the actuator.

Parameter	Symbol	Value
Chamber diameter	*d*	31.5 mm
Chamber height	*h*	4 mm
Piezoelectric oscillator substrate diameter	*D_p_*	35 mm
Piezoelectric oscillator ceramic diameter	*d_p_*	25 mm
Driving voltage	*U*	100 V
